# Recombinant production and initial characterization of a novel lectin-like protein from the red alga *Kappaphycus alvarezii*

**DOI:** 10.1016/j.biotno.2026.09.001

**Published:** 2026-09-30

**Authors:** Éllen Francine Rodrigues, Gabrielly Oliveira do Couto, Lauane Ferreira de Almeida, Ana Lúcia Fachin, Renê de Oliveira Beleboni, Mozart Marins

**Affiliations:** a Biotechnology Unit, University of Ribeirão Preto, Ribeirão Preto, São Paulo, Brazil; b Postgraduate Program in Environmental Technology, University of Ribeirão Preto, Ribeirão Preto, São Paulo, Brazil; c Algastech Aquiculture, Research and Development, Ubatuba, São Paulo, Brazil

**Keywords:** Marine macroalgae, Recombinant lectin, Carbohydrate-binding protein, Osteosarcoma, Anticancer activity

## Abstract

Marine macroalgae are essential sources of bioactive compounds, widely explored for their pharmacological and biotechnological properties. *Kappaphycus alvarezii* (Rhodophyta), a red macroalga widely cultivated worldwide as a major source of κ-carrageenan, is emerging as a rich source of lectins with high carbohydrate-binding specificity, offering promising applications in diagnostics and antitumor therapies. This study aimed to recombinantly produce, purify, and characterize the biological activities of a novel lectin-like protein from *K. alvarezii*, designated KAL-1 lectin-like, with emphasis on its anticancer potential against human osteosarcoma U2OS cells. The recombinant KAL-1 lectin-like protein was expressed in CHO cells, purified by affinity chromatography, and subsequently evaluated for cytotoxicity and cellular selectivity using the MTT viability assay in HaCaT and U2OS cell lines. KAL-1 significantly reduced the viability of U2OS cells at very low concentrations (0.025 μM), without inducing relevant inhibitory effects on the viability of normal epithelial cells (HaCaT). These results indicate a selective antitumor action of KAL-1, suggesting its potential as a promising candidate for the development of targeted anticancer agents.

## 1 Introduction

Cancer represents one of the most significant global public health challenges, with its incidence having increased by approximately 20% over the past decade. By 2030, it is estimated that more than 23.6 million new cases will be diagnosed worldwide.^1^ This heterogeneous group of over one hundred diseases is characterized by genetic alterations that promote uncontrolled cell proliferation and survival.^2^ Among the mechanisms involved in carcinogenesis, genomic instability and deficiencies in DNA damage response pathways stand out, as they contribute to the accumulation of mutations and tumor progression.^3^ Another critical aspect of cancer development involves the abnormal glycosylation of cell-surface glycoconjugates, which disrupts processes such as cell recognition, communication, and adhesion.^4^^,^^5^ Given this complexity, the search for bioactive compounds that selectively interact with glycan structures associated with tumor cells has emerged as a promising strategy for the development of new anticancer therapies.^6^

In this context, marine macroalgae have gained attention as natural sources of structurally diverse biomolecules with significant biological potential. Traditionally consumed for millennia in various Eastern cultures for their nutritional and medicinal properties, these organisms have increasingly attracted scientific interest in recent decades. Studies have demonstrated that bioactive metabolites derived from macroalgae benefit human health, highlighting their potential as novel therapeutic and pharmaceutical product sources.^7^^,^^8^ Algae are eukaryotic, photosynthetic organisms that predominantly inhabit coastal environments and are promising natural sources of secondary metabolites with broad biological activities.^9^ Based on pigmentation and chemical composition, the macroalgae are classified into three main phyla: Chlorophyta (green algae), Phaeophyta (brown algae), and Rhodophyta (red algae).^10^ Among these, red algae are particularly notable for their economic importance and high levels of bioactive compounds.^11^

The red alga *Kappaphycus alvarezii* has attracted increasing scientific and industrial interest due to its remarkable biotechnological potential. Originally from the Philippines, it is cultivated in several tropical regions as a significant raw material for the production of carrageenan, particularly the kappa type.^12^ This sulfated polysaccharide has wide-ranging applications in the food, pharmaceutical, and cosmetic industries, where it functions as a stabilizing, thickening, emulsifying, and gelling agent.^13^ In Brazil, *K. alvarezii* was introduced experimentally about three decades ago in Ubatuba, São Paulo, to meet the growing demand for carrageenan, which had previously been supplied exclusively by natural stocks of the native species *Hypnea musciformis* and by imports.^14^ Since its introduction, numerous studies have revealed that this macroalga exhibits a broad spectrum of biological activities, including antioxidant,^15^^,^^16^ anticancer,^16^ antimicrobial,^16^ anti-inflammatory,^17^ antifungal,^18^ and antiviral properties. ^19^

Lectins have drawn particular scientific attention among the bioactive metabolites identified in *K. alvarezii*. Lectins are carbohydrate-binding proteins capable of recognizing and binding specifically to glycan structures on the surfaces of cells and microorganisms. Red algal lectins are of particular interest due to their high affinity for glycans typically expressed on tumor cells, viruses, and pathogens, making them promising candidates for drug development and targeted therapies.^20^^,^^21^ Recent analyses of the Rhodophyta phycolectome further highlight red algal lectins as versatile biotechnological tools, including potential biomarkers, biosensors, drug-delivery platforms, and lead molecules for anticancer and antimicrobial drugs.^22^ Several lectins from red algae have already demonstrated significant anticancer effects, and some share high structural similarity with those from *K. alvarezii*.^23^

Nevertheless, despite their biotechnological potential, few reports are available in the literature on lectins derived from *K. alvarezii*. The Siye lectin, isolated from this species, has demonstrated significant antitumor activity, inhibiting the growth of human lung carcinoma (A549) and breast adenocarcinoma (HCC1937) cells.^24^ Sequence analysis revealed a high degree of homology between Siye and the previously described lectins KAA-1 and KAA-2, suggesting an evolutionary advantage related to their ability to recognize high-mannose structures. Lectins isolated from the red algae *Eucheuma serra* (ESA-2) and *K. alvarezii* (KAA-2), as well as from the green alga *Boodlea coacta* (BCA), have also exhibited strong anti-HIV and anti-influenza activities.^25^^,^^26^ Furthermore, a novel lectin isolated from the marine red alga *Meristiella echinocarpa* (MEL) demonstrated the ability to recognize and agglutinate pathogenic bacterial strains, including multidrug-resistant *Salmonella* and *Vibrio alginolyticus*,^27^ reinforcing the broad antimicrobial potential of macroalgal lectins.

Complementarily, studies on *K. striatus* identified another lectin (KSL) with anticancer properties and substantial sequence similarity to other high-mannose-binding lectins, including ESA-2, KAA-1, KAA-2, Sfl-1, and Sfl-2.^28^ This structural homology suggests that KSL may share binding and recognition mechanisms, exhibiting high affinity for mannose-rich glycans on tumor cell surfaces, thereby enhancing selective apoptotic effects. In addition to their anticancer, antiviral, and antioxidant activities, several macroalgal lectins exhibit hemagglutinating and immunomodulatory properties, being capable of modulating cell signaling pathways, inducing apoptosis, and influencing DNA repair processes.^29^^,^^30^ Nevertheless, despite these advances, the mechanisms of action, molecular specificity, and therapeutic potential of lectins isolated from *K. alvarezii* remain poorly understood.

To address this gap, we recombinantly expressed a novel marine lectin-like protein (KAL-1) from *K. alvarezii* in Chinese hamster ovary (CHO) cells and evaluated its selective antitumor activity against human osteosarcoma (U2OS) cells and its effects on cell viability in normal keratinocyte (HaCaT) cells, providing an initial contribution to the emerging characterization of *K. alvarezii* lectins.

## 2 Material and methods

### 2.1 Identification of lectin-like sequence IN *K. Alvarezii*

Putative lectin homologs in *Kappaphycus alvarezii* were identified by performing TBLASTN searches against the *K. alvarezii* genome assembly (GCA_002205965.3; ASM220596v3) using a curated dataset of lectin protein sequences from *Rhodophyta* as queries. Previously characterized *Kappaphycus* lectins, such as KAA-1 and KAA-2, were excluded to prevent redundancy. Among the resulting hits, a locus corresponding to *K. alvarezii* cultivar kp tig00000488 (whole-genome shotgun sequence ID: NADL03000813.1) exhibited strong similarity to a lectin-like protein from *Gracilaria fisheri* (accession ACY56710.1; E-value = 2 × 10^−151^). The genomic region encompassing this locus was extracted and analyzed using ORFfinder (NCBI) to predict putative open reading frames. BLASTP was subsequently used to analyze the predicted protein sequences against the NCBI non-redundant (nr) database, restricted to *Rhodophyta* (taxid: 2763), which confirmed their similarity to lectin proteins and revealed a conserved L-type (legume-type) lectin domain. This newly identified *Kappaphycus* lectin-like protein was designated KAL-1. All BLAST analyses were performed using BLAST + v2.17.0 with default parameters, and the NCBI nr database was accessed in July 2025 (Fig. 1).

**Fig. 1:**
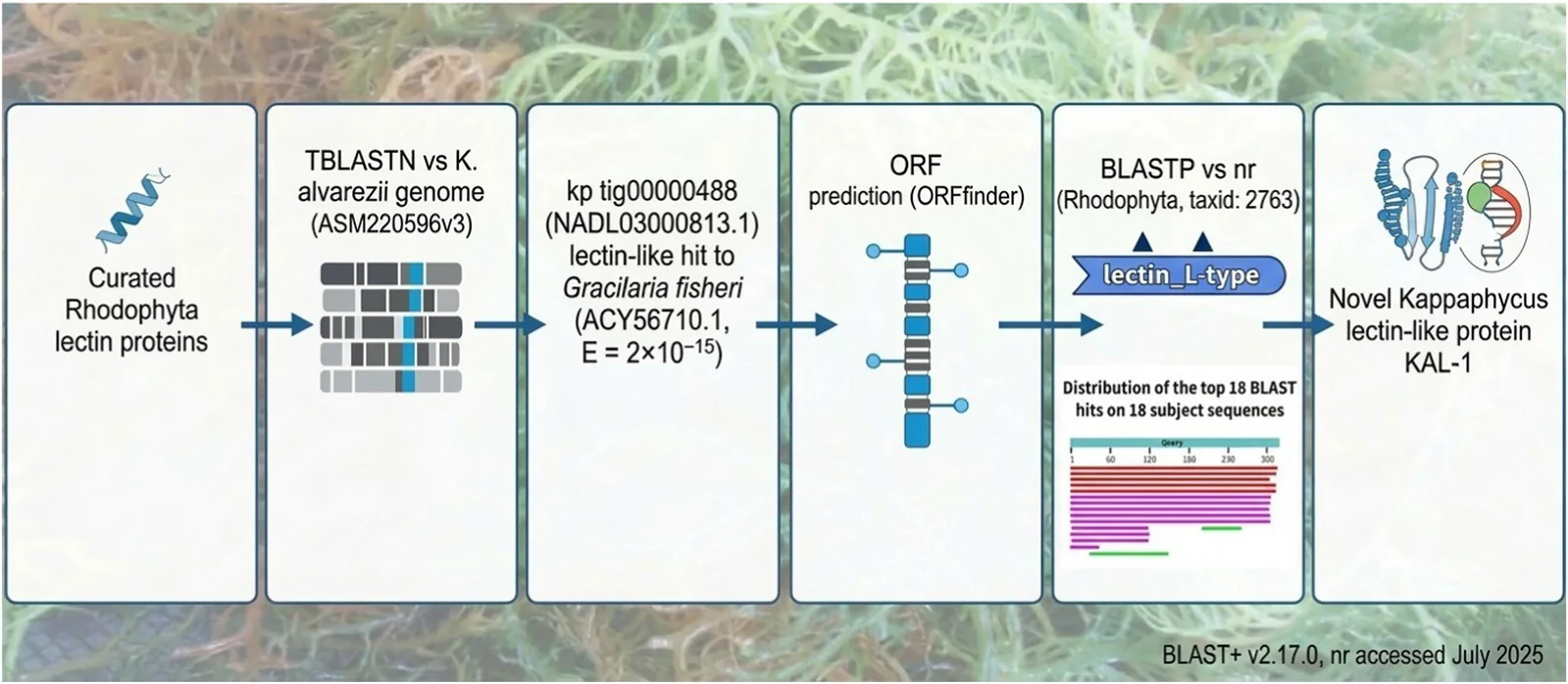
Bioinformatic workflow for the identification of the novel lectin-like protein KAL-1 in *K*. *alvarezii*, based on TBLASTN screening of curated Rhodophyta lectin sequences against the genome, followed by ORF prediction and BLASTP analysis against the non-redundant Rhodophyta protein database. Nucleotide and deduced amino acid sequences of Kal-1. The predicted legume-like lectin domain is indicated by underlined amino acids. The stop codon is marked by an asterisk (*).

### 2.2 KAL-1 lectin recombinant production

The complete sequence of the KAL-1 lectin from *K. alvarezii* was elucidated by peptide mapping and complementary DNA (cDNA) cloning. Lectin identification was based on database analyses from the *K. alvarezii* genome and transcriptome sequencing projects.

The mammalian CHO-S (Chinese Hamster Ovary Suspension) expression system was selected after unsuccessful expression of the KAL-1 lectin in *Escherichia coli*, where the protein was obtained mainly as insoluble inclusion bodies. Since lectin activity depends on proper folding and disulfide bond formation, a mammalian system was chosen to provide an appropriate cellular environment for protein folding, structural stability, and the recovery of a soluble, biologically active recombinant protein.

Recombinant KAL-1 lectin expression was performed by GenScript (Piscataway, NJ, USA). Briefly, the target DNA sequence encoding KAL-1 was designed, codon-optimized for mammalian expression, chemically synthesized, and subcloned into a 7066-bp mammalian expression vector (Fig. 2). The construct contained a CMV enhancer/promoter for high-level transcription, a secretion signal sequence, the KAL-1 coding region fused to a C-terminal 6×His tag to facilitate purification, a woodchuck hepatitis virus post-transcriptional regulatory element (WPRE), and a poly(A) signal. In addition, the plasmid harbored ampicillin and neomycin/kanamycin resistance genes, as well as bacterial and f1 origins of replication. Transfection-grade plasmid DNA was obtained by maxi-preparation prior to expression.

**Fig. 2:**
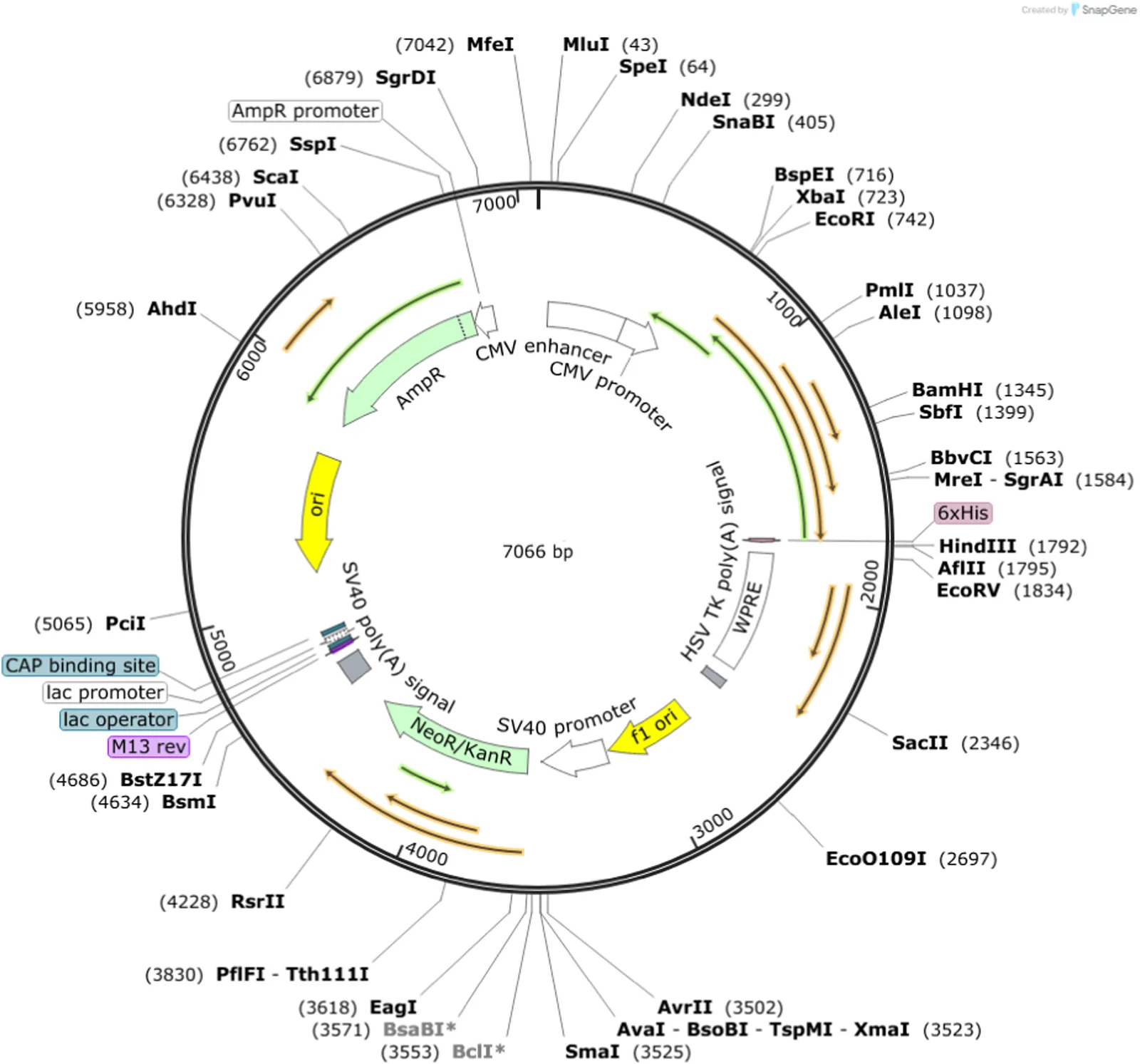
Schematic representation of the mammalian expression vector used for recombinant production of KAL-1 lectin in CHO-S cells, highlighting the key functional elements including the promoter driving transgene expression, signal peptide sequence, KAL-1 coding region, selection marker, and regulatory elements required for stable expression and protein production in the CHO-S mammalian expression system. The 7066-bp plasmid contains a CMV enhancer/promoter, secretion signal peptide, KAL-1 coding sequence fused to a C-terminal 6×His tag, WPRE element, poly(A) signal, ampicillin (AmpR) and neomycin/kanamycin (NeoR/KanR) resistance genes, and bacterial and f1 origins of replication.

The protein was expressed in CHO-S (Chinese Hamster Ovary Suspension) cells cultured in MB C1 Medium (GenScript) supplemented with GenScript Enhancer, GenScript Feed A, and GenScript Feed B. Cells were maintained at 37 °C under 5% CO_2_ with orbital agitation, and one day before transfection, they were seeded at an appropriate density. On the day of transfection, plasmid DNA and transfection reagent were mixed at an optimized ratio and added to the cells. Approximately 24 h post-transfection, feed supplements were added to support protein production. The culture was initially maintained at 37 °C, then reduced to 32 °C on Day 1 to improve protein yield and quality. After a total expression period of three days, the culture supernatant containing the secreted recombinant lectin was collected for purification.

The recombinant KAL-1 lectin was purified using PreDictor RoboColumn IMAC Sepharose 6FF (GenScript, USA), taking advantage of the C-terminal 6× His tag. After affinity chromatography, the eluted fractions were pooled and buffer-exchanged to the final formulation buffer. The purified protein was analyzed by CE-SDS/SDS-PAGE and size-exclusion high-performance liquid chromatography (SEC-HPLC) to determine molecular weight and purity, and protein concentration was determined spectrophotometrically by the A280 method.

#### 2.2.1 SDS-PAGE

SDS-PAGE was used to analyze protein samples under reducing and non-reducing conditions to assess their subunit composition. Molecular weights were estimated using Bio-Rad protein standards (Cat. No. 1610374S).

#### 2.2.2 Size-exclusion chromatography

To assess their molecular weight distribution, protein samples were characterized by size-exclusion chromatography (SEC). Analyses were conducted using an H-Class Bio system (GR23010364) with a Phenomenex Biozen column (Column 376#). The mobile phase consisted of 0.1 M Na_2_SO_4_ in 0.118 M phosphate buffer (pH 6.7 ± 0.3). For each run, 40 μL of sample was injected into the column, which had been pre-equilibrated with the mobile phase. Elution was monitored at 280 nm, and chromatograms were recorded over 8 min. Molecular weight calibration was performed using standard proteins of known molecular weights, allowing estimation of the apparent molecular weights of sample peaks from their retention times. Chromatographic peak parameters, including retention time, height, width, area, and relative area percentage, were determined using the instrument's data analysis software. Individual protein species were identified and quantified, enabling evaluation of sample composition and purity.

### 2.3 Biological activity

#### 2.3.1 Cell lines and culture conditions

Cell viability assays were performed using HaCaT (keratinocytes) and U2OS (osteosarcoma) cell lines. HaCaT cells were cultured in RPMI medium (10.4 g/L RPMI, 2.0 g/L NaHCO_3_, 5.95 g/L HEPES) supplemented with 10% fetal bovine serum (Cultilab), while U2OS cells were maintained in McCoy's medium (12.5 g/L McCoy, 2.2 g/L NaHCO_3_) with 10% FBS. Both cell lines were incubated at 37 °C in a humidified atmosphere containing 5% CO_2_. Culture media were supplemented with amphotericin B (1.25 μg/mL), kanamycin (50 μg/mL), penicillin (50 U/mL), combined with streptomycin (50 μg/mL), and ciprofloxacin (10 μg/mL). Media were replaced every 48 h, and cells reaching ∼90% confluence were subcultured by trypsinization.

#### 2.3.2 Cell viability

Cell viability was evaluated using the MTT assay [3-(4,5-dimethylthiazol-2-yl)-2,5-diphenyltetrazolium bromide]^31^ in HaCaT and U2OS cells. Cells were seeded in flat-bottomed 96-well plates at a density of 1 × 10^4^ cells/mL in 200 μL of medium and incubated at 37 °C with 5% CO_2_ for 24 h to allow adhesion. Afterward, cells were treated with four concentrations of KAL-1 lectin (0.025 μM, 0.050 μM, 0.100 μM, and 0.200 μM). As a positive control, the commercial chemotherapeutic agent doxorubicin (doxorubicin hydrochloride, Sigma-Aldrich CAS. 25316-40-9) was used at the same concentrations. Cells were incubated with the treatments for 48 h to assess the effects on cell viability in normal and tumor cell lines. Cell viability was then determined using the MTT assay (Sigma-Aldrich, CAS 298-93-1). Experiments were performed independently three times, with duplicates for each condition. Optical density (OD) was measured at 550 nm using a Thermo Scientific Multiskan FC microplate photometer.

#### 2.3.3 Statistical analysis

Statistical analyses were performed using GraphPad Prism software. Data were analyzed by one-way analysis of variance (ANOVA) followed by Tukey's multiple comparisons test. Differences were considered statistically significant at p < 0.05.

## 3 Results and discussion

### 3.1 KAL-1 lectin-like recombinant production

Bands around 50 kDa were observed under both reducing (R) and non-reducing (NR) conditions (Fig. 3). Comparison of the R and NR conditions indicates that the protein maintains similar electrophoretic mobility in both conditions. No higher-molecular-weight bands were detected in the non-reducing gel, indicating the absence of intermolecular disulfide-bond-mediated oligomerization. This suggests that cysteine residues do not form intermolecular disulfide bonds (cystine), leading to SDS-PAGE-detectable aggregates. Nevertheless, the presence of intramolecular disulfide bonds cannot be excluded, as they would not alter the apparent molecular weight under denaturing conditions. Furthermore, the lack of multiple lower-molecular-weight bands or signs of degradation indicates that the recombinant protein remains stable during expression and harvesting, with no significant proteolytic cleavage. The concentration of KAL-1 lectin-like expressed was 30 μg/mL.

**Fig. 3:**
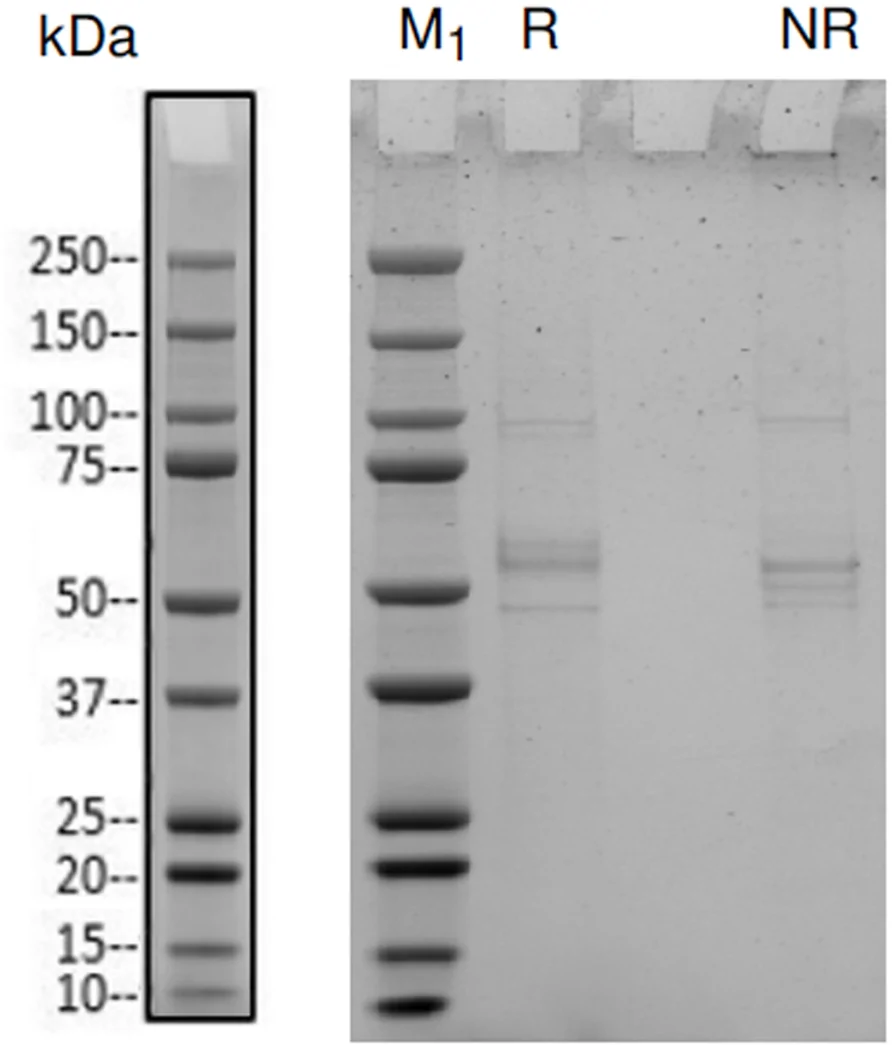
SDS-PAGE analysis of the purified protein under reducing (R) and non-reducing (NR) conditions. Lane M_1_ corresponds to the molecular weight marker (10–250 kDa), while lanes R and NR represent the protein samples analyzed under reducing and non-reducing conditions, respectively. Molecular masses (kDa) are indicated on the left.

The chromatogram (Fig. 4) shows multiple peaks over the retention time (RT) range of 2.6 to 4.3 min, indicating a heterogeneous sample with an apparent molecular mass. When comparing the retention times of the sample peaks with those of the standards (gray line), it is observed that the primary signal, with an RT of 3.5 min and accounting for 33% of the total area, corresponds to an approximate molecular mass of 150–160 kDa, suggesting the predominance of an oligomeric or aggregated form of the recombinant protein. In addition, other relevant peaks were detected at RT 2.6 min (12% of the area), 3.1 min (11%), and 4.0 min (17%). The peaks with shorter retention times (2.6–3.1 min) correspond to high-molecular-weight species, possibly aggregates or complex macromolecular assemblies formed during expression or purification. Conversely, the peak at 4.2 min, which coincides with the 44 kDa standard, represents only about 11% of the total area and may be attributed to the monomeric form of the recombinant protein. The less intense signals at higher retention times (RT > 4.2 min) may be associated with proteolytic degradation fragments or minor co-eluting impurities.

**Fig. 4:**
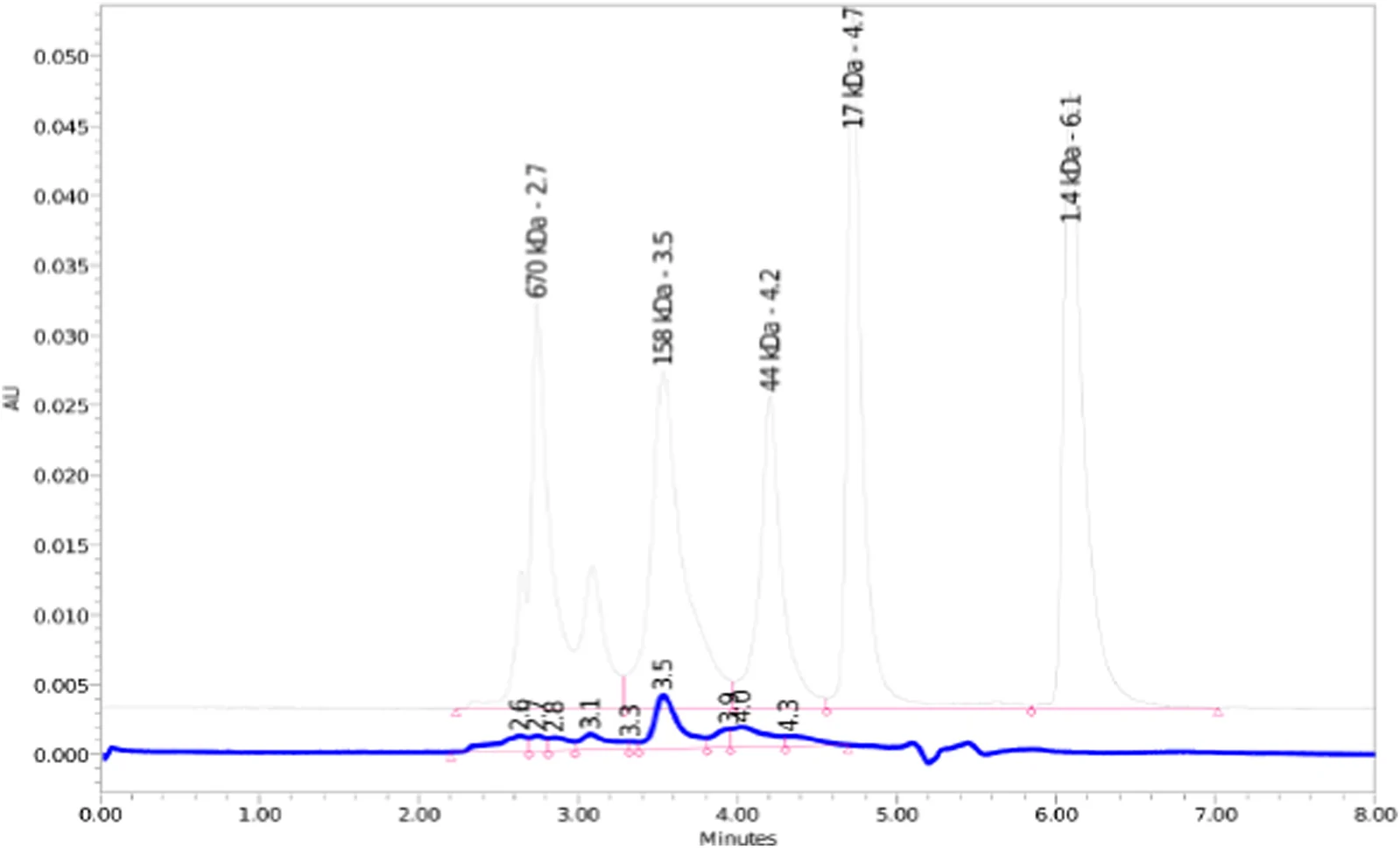
HPLC size-exclusion chromatography (HPLC-SEC) profile of purified recombinant KAL-1 lectin-like protein. The blue trace represents the protein sample, while the gray trace corresponds to the molecular weight standards used for column calibration. The calibration curve includes standards with apparent molecular masses of 670, 158, 44, 17, and 1.4 kDa, with their respective retention times indicated above each peak.

The recombinant KAL-1 lectin-like protein exhibited an apparent molecular mass of approximately 50 kDa under both reducing and non-reducing conditions. In contrast, lectins previously isolated from *K*. *alvarezii*, including KAA-1 (267 amino acids; 27 kDa) and KAA-2 (268 amino acids; 28 kDa), have been reported to migrate at approximately 28 kDa in SDS-PAGE under both reducing and non-reducing conditions.^24^ The discrepancy suggests that recombinant expression in a heterologous system may influence folding efficiency, post-translational processing, or oligomerization behavior of KAL-1.

Taken together, the SDS-PAGE and SEC-HPLC data provide a coherent model for the structural organization of recombinant KAL-1. SDS-PAGE indicates a stable species of approximately 50 kDa under both reducing and non-reducing conditions, while SEC-HPLC reveals that, under native conditions, the protein predominantly assembles into a higher-order species of approximately 150–160 kDa. These observations suggest that the functional building block of KAL-1 in solution is not the theoretical 28 kDa monomer but rather a higher-mass subunit in the range of 50–60 kDa. Within this framework, the dominant SEC peak at 150–160 kDa is consistent with a trimeric assembly of this subunit, indicating that this oligomeric form represents the major native species in solution. The presence of additional higher-molecular-weight peaks between 2.6 and 3.3 min likely reflects further oligomerization or non-specific aggregation of the same structural unit, whereas the minor peak at 4.0–4.2 min may correspond to a less assembled or partially processed form of the protein.

Overall, the data support a model in which recombinant KAL-1 forms a ∼50–60 kDa structural subunit that predominantly assembles into a ∼150–160 kDa trimeric complex in solution, with additional minor oligomeric and partially processed species present. The relatively low expression yield (30 μg/mL) further suggests that folding efficiency or post-translational maturation may be suboptimal under the current expression conditions, a known challenge for heterologous production of lectins and other structurally constrained proteins.

Further clarification of the molecular organization of KAL-1 could benefit from complementary orthogonal approaches. Intact mass spectrometry or peptide mapping could help elucidate whether the ∼50 kDa species corresponds to a post-translationally modified monomer, a tag-containing construct, or an SDS-stable assembly. Similarly, dynamic and multi-angle light scattering could provide additional support for the oligomeric state and offer a more accurate estimate of the absolute molecular mass of the native species observed by SEC. Circular dichroism spectroscopy could also contribute to a better understanding of secondary-structure content and overall folding quality, potentially aiding the distinction between functional oligomerization and aggregation associated with incomplete folding during heterologous expression.

### 3.2 Biological activity

Cell viability was evaluated using the MTT assay after 48 h of exposure to the KAL-1 lectin-like compound in HaCaT and U2OS cell lines. Distinct behaviors were observed between the cell lines, indicating a possible biological selectivity of the protein. In HaCaT cells, the KAL-1 lectin-like compound did not reduce cell viability at any tested concentration (0.025–0.200 μM), with viability values exceeding 100% in all treatments. The observed increase, ranging from 106.15% to 122.31%, suggests that the compound may enhance metabolic or mitochondrial activity, possibly indicating a trophic effect related to the activation of metabolic pathways associated with cell survival. Similar findings have been reported for lectins and recombinant proteins capable of modulating mitochondrial activity and gene expression involved in maintaining cellular integrity.^32^

In contrast, the U2OS tumor cell line exhibited a progressive reduction in viability as the concentration of the KAL-1 lectin-like compound increased, from 68.44% to 57.33%. This dose-dependent profile suggests a potential selective effect on tumor cell viability, possibly associated with the induction or inhibition of apoptosis or the inhibition of proliferative signaling pathways. Lectins with affinity for specific glycoconjugates in malignant cells often display this behavior, promoting programmed cell death or suppression of tumor metabolism.^30^^,^^33^

Overall, KAL-1 lectin-like did not affect keratinocyte viability while exerting a concentration-dependent reduction in osteosarcoma cell viability, suggesting a promising selective action for antitumor applications. Further investigations, including apoptosis assays, cell cycle analysis, and evaluation of molecular marker expression, are required to elucidate the mechanisms underlying this differential response. Within the concentration range evaluated (0.025–0.200 μM, 48 h), KAL-1 lectin-like did not reduce HaCaT viability, which remained above 100% at all concentrations, and U2OS viability did not fall below approximately 57–60% of control; therefore, LC50/IC50 values could not be determined under the present experimental conditions. These findings should be interpreted as preliminary evidence of selective antiproliferative activity rather than a complete pharmacological characterization.

Fig. 5 shows the evaluation of cell viability obtained through the MTT assay in HaCat and U2OS cell lines after treatment with different concentrations of KAL-1 lectin-like and doxorubicin. Fig. 5a shows that treatment with KAL-1 lectin-like significantly decreased the viability of the tumor cell line (U2OS) in a concentration-dependent manner. While HaCat cells maintained viability close to 100%, U2OS cells exhibited a marked reduction at all tested concentrations, particularly at 0.100 and 0.200 μM, where viability decreased to approximately 57–60%. These findings indicate a higher tumor cell line (U2OS) sensitivity to KAL-1 KAL-1 lectin-like treatment, suggesting a potential selective effect on cancer cell viability.

**Fig. 5:**
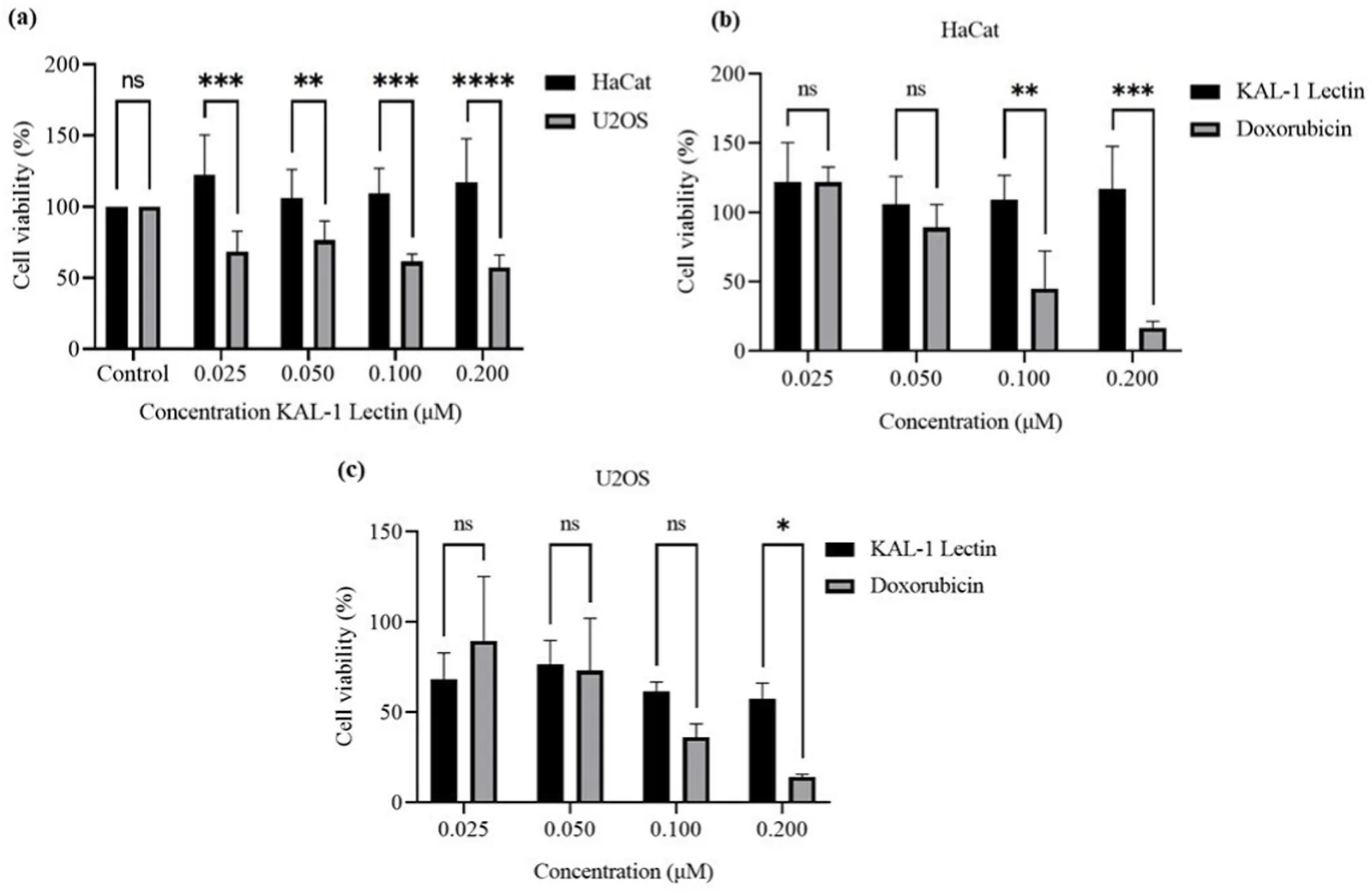
Cell viability determined by the MTT assay after treatment with different concentrations of recombinant KAL-1 lectin-like and doxorubicin. (a) Comparison of the viability of HaCat and U2OS cells after exposure to KAL-1 lectin-like (0.025–0.200 μM). (b) Comparison between KAL-1 lectin-like and doxorubicin treatments in HaCat cells. (c) Comparison between KAL-1 lectin-like and doxorubicin treatments in U2OS cells. Cell viability is expressed as a percentage relative to the untreated control. Data are presented as mean ± standard deviation. Statistical significance is indicated as ns, not significant; *p < 0.05, **p < 0.01, ***p < 0.001, and ****p < 0.0001.

The selective cytotoxicity observed for the KAL-1 lectin-like, characterized by a significant reduction in the viability of the U2OS tumor cell line while preserving the viability of HaCat cells, is consistent with profiles reported for several lectins of plant, fungal, and algal origin. Eutirucallin, isolated from *Euphorbia tirucalli*, reduced the viability of different tumor cell lines without affecting fibroblasts and macrophages,^34^ whereas the lectins SRL from *Sclerotium rolfsii*, VML from *Vatairea macrocarpa*, and RBL from *Rhizoctonia bataticola* also exhibited preferential cytotoxic activity against neoplastic cells, with limited effects on normal cells.^35^, ^36^, ^37^ These findings suggest that the selectivity observed for KAL-1 lectin-like represents a recurrent feature among lectins with antitumor potential and is frequently associated with the recognition of glycans differentially expressed on the surface of malignant cells.

Furthermore, the KAL-1 lectin-like and its biological activity at low micromolar concentrations place this protein within the context of high-mannose-binding algal lectins (OAAH), which have been described in species of the genus *Kappaphycus*. The lectin KSL, isolated from *K*. *striatus*, exhibited antiproliferative activity against several carcinoma cell lines (HT29, HeLa, MCF-7, SK-LU-1, and AGS), with IC_50_ values ranging from 0.80 to 1.94 μM.^38^ Similarly, a homologous lectin identified in *K*. *alvarezii* demonstrated antitumor activity against breast adenocarcinoma (HCC1937), lung carcinoma (A549), liver cancer (HepG2), and promyelocytic leukemia (HL60) cell lines.^24^ Considering that KAL-1 induced a marked reduction in cell viability at concentrations ranging from 0.025 to 0.200 μM, the present results indicate a high biological potency when compared with related algal lectins. Likewise, the mannose-specific lectin BPL2, isolated from *Bryopsis plumosa*, exhibited dose- and time-dependent cytotoxicity against lung cancer cell lines (A549, H460, and H1299), while showing low toxicity toward non-tumor cells.^39^ Collectively, these findings support the hypothesis that the recognition of mannose-rich glycans, which are often present at higher densities on the surface of tumor cells, may represent one of the main mechanisms underlying the selective cytotoxicity of KAL-1.

Fig. 5b and c shows that, compared with the doxorubicin control, KAL-1 lectin-like exhibited no detectable reduction in cell viability in HaCat cells at all concentrations (0.025–0.200 μM). This result highlights the lectin's higher biocompatibility with non-tumoral cells, a desirable feature for compounds considered potential therapeutic agents. Conversely, in the U2OS cell line (Fig. 5c), the lectin reduced cell viability to levels comparable to doxorubicin at lower concentrations, with a significant difference observed only at 0.200 μM (p < 0.05), where doxorubicin showed a more potent effect. Nevertheless, the decreasing trend in viability with increasing lectin concentration confirms its dose-dependent action and potential antiproliferative effect on tumor cells. This finding is particularly relevant because KAL-1 maintained a selective cytotoxic profile, preserving the viability of non-tumor cells while exerting pronounced antiproliferative activity against tumor cells. Strategies based on this property have been explored to enhance the selectivity of conventional chemotherapeutic agents. In this context, lectin-mediated pretargeting systems associated with doxorubicin have demonstrated lower cytotoxicity toward normal cells compared with free doxorubicin, while maintaining or even enhancing antitumor efficacy in cancer cell lines.^40^ These findings support the hypothesis that lectins with affinity for tumor-associated glycans may function both as selective cytotoxic agents and as targeting molecules for antineoplastic drugs, thereby increasing therapeutic specificity and reducing systemic adverse effects.

Nevertheless, the magnitude of the effect observed in the present study, with cell viability reduced to approximately 57–60% at the highest concentrations tested, can be considered moderate when compared with some lectins reported in the literature. For example, MytiLec-1, isolated from the mussel *Mytilus galloprovincialis*, exhibited dose-dependent antiproliferative activity against U937 and HeLa cells, inhibiting cell growth by 36% to 63% at concentrations ranging from 12.5 to 50 μg/mL.^41^ Although direct comparisons are limited by differences in cell models, experimental conditions, and concentration units, the present results demonstrate that KAL-1 lectin-like possesses measurable and selective cytotoxic activity against U2OS cells. Future studies with a broader concentration range and more detailed characterization of the dose-response relationship will improve the accuracy of its antitumor potency assessment and enable more robust comparisons with other bioactive lectins reported in the literature.

KAL-1 lectin exhibits selective effects on cell viability, inhibiting U2OS cell proliferation while preserving HaCaT cell viability. This behavior may be associated with the carbohydrate-recognition specificity characteristic of lectins, which can influence cellular adhesion, signaling, and programmed cell death (apoptosis) in malignant cells. Therefore, the data indicate the biotechnological potential of the KAL-1 lectin-like protein as an antitumor agent, capable of acting selectively and with lower toxicity than conventional drugs such as doxorubicin. An important limitation of the present study is that, within the tested concentration range (0.025–0.200 μM), KAL-1 lectin-like did not reach 50% inhibition of U2OS viability, which precluded reliable calculation of LC50/IC50 values. In addition, due to the limited amount of recombinant protein available from this initial production and the exploratory nature of this short communication, we were unable to perform mechanistic assays of cell death, such as annexin V/PI staining, caspase activation, TUNEL, or analysis of autophagy markers. Our data, therefore, provide proof-of-concept evidence of selective antiproliferative activity against osteosarcoma cells and reinforce the need for further studies to elucidate its mechanisms of action and cellular selectivity, with full concentration–response characterization and detailed investigation of the underlying apoptotic or autophagic pathways.

The osteosarcoma cell line was selected for this study based on growing evidence that lectins exhibit selective anticancer activity through the recognition of altered glycosylation patterns commonly found in tumor cells. Although no previous studies have directly evaluated algae- or seaweed-derived lectins against osteosarcoma cells, several marine lectins from red algae, such as those isolated from *Eucheuma serra*, *Solieria filiformis*, and *K*. *striatus*, have demonstrated potent cytotoxic and apoptosis-inducing activity against multiple cancer cell types, including solid tumors and leukemia cells. These lectins are known to recognize high-mannose glycans and other abnormal glycan structures that are frequently overexpressed in malignant cells.^25^^,^^38^^,^^42^ Osteosarcoma cells present significant alterations in glycosylation profiles, including abnormal N-glycans and fucosylated glycoproteins, which have already been explored using lectin-based glycomic analyses to identify metastatic phenotypes.^43^ In addition, the mechanisms commonly associated with marine lectin-mediated cytotoxicity, including caspase-dependent apoptosis, autophagic cell death, membrane disruption, and glycan-specific internalization, are highly relevant to osteosarcoma biology.^44^ Previous studies also demonstrated antitumor effects of lectins in sarcoma models, such as sarcoma-180, reinforcing the rationale for investigating lectin activity in osteosarcoma cells.^45^ Therefore, the use of osteosarcoma cells in the present study was based on their biological susceptibility to lectin-glycan interactions and the potential of marine lectins as novel anticancer agents targeting tumor-associated glycans.

## 4 Conclusions

The results demonstrated distinct cellular responses, suggesting selective biological activity of the KAL-1 lectin-like. In HaCaT cells, the lectin did not reduce cell viability at any tested concentration (0.025–0.200 μM), with cell viability exceeding 100%, suggesting a potential proliferative or trophic effect, likely associated with the activation of metabolic pathways supporting cell survival. In contrast, U2OS cells showed a concentration-dependent reduction in viability, consistent with a selective effect on tumor cell viability. Comparative analysis with the control (doxorubicin) further confirmed the selective profile of the KAL-1 lectin-like. While HaCaT cells maintained viability close to 100% under KAL-1 lectin-like treatment, a significantly smaller reduction in cell viability was observed than with doxorubicin, highlighting the lectin's higher biocompatibility with non-tumoral cells. In U2OS cells, KAL-1 lectin-like reduced viability dose-dependently, achieving effects comparable to doxorubicin at lower concentrations and demonstrating potential antiproliferative activity.

Osteosarcoma is the most common malignant bone tumor in children and adolescents and still presents poor outcomes in metastatic or recurrent cases, highlighting the need for novel targeted therapeutic agents. ^46^ Although our in vitro data represent an early proof of concept and require validation in additional models, the KAL-1 lectin-like showed promising selective antitumor activity against osteosarcoma U2OS cells while maintaining high viability in non-tumoral cells, supporting its biotechnological potential as a candidate therapeutic agent. Future work will focus on increasing recombinant KAL-1 yield to enable extended dose–response assays for LC50/IC50 determination and detailed investigation of potential KAL-1-induced cell-death mechanisms in osteosarcoma models.

## CRediT authorship contribution statement

**Éllen Francine Rodrigues:** Writing – review & editing, Writing – original draft, Supervision, Methodology, Investigation, Data curation. **Gabrielly Oliveira do Couto:** Methodology. **Lauane Ferreira de Almeida:** Writing – original draft, Methodology, Data curation. **Ana Lúcia Fachin:** Visualization, Resources. **Renê de Oliveira Beleboni:** Visualization, Resources. **Mozart Marins:** Writing – review & editing, Supervision, Resources, Project administration, Conceptualization.

## Funding statement

The authors acknowledge the financial support from the Coordination for the Advancement of Higher Education Personnel (10.13039/501100002322CAPES), the 10.13039/501100003593National Council for Scientific and Technological Development (10.13039/501100003593CNPq), and the 10.13039/501100008353University of Ribeirão Preto.

## Declaration of competing interest

The authors declare that they have no known competing financial interests or personal relationships that could have appeared to influence the work reported in this paper.
